# Censorship and Suppression of Covid-19 Heterodoxy: Tactics and Counter-Tactics

**DOI:** 10.1007/s11024-022-09479-4

**Published:** 2022-11-01

**Authors:** Yaffa Shir-Raz, Ety Elisha, Brian Martin, Natti Ronel, Josh Guetzkow

**Affiliations:** 1 Department of Communication, University of Haifa, Haifa, Israel; 2 Raphael Recanati International School, IDC, Herzliya, Israel; 3 Department of Criminology, The Max Stern Yezreel Valley College, Jezreel Valley, Israel; 4 Humanities and Social Inquiry, University of Wollongong, Wollongong, Australia; 5 Department of Criminology, Bar Ilan University, Ramat Gan, Israel; 6 Department of Sociology and Anthropology, Institute of Criminology, The Hebrew University of Jerusalem, Jerusalem, Israel

**Keywords:** COVID-19, Censorship, Suppression of dissent, Scientific debate, Ethics, Public health

## Abstract

The emergence of COVID-19 has led to numerous controversies over COVID-related knowledge and policy. To counter the perceived threat from doctors and scientists who challenge the official position of governmental and intergovernmental health authorities, some supporters of this orthodoxy have moved to censor those who promote dissenting views. The aim of the present study is to explore the experiences and responses of highly accomplished doctors and research scientists from different countries who have been targets of suppression and/or censorship following their publications and statements in relation to COVID-19 that challenge official views. Our findings point to the central role played by media organizations, and especially by information technology companies, in attempting to stifle debate over COVID-19 policy and measures. In the effort to silence alternative voices, widespread use was made not only of censorship, but of tactics of suppression that damaged the reputations and careers of dissenting doctors and scientists, regardless of their academic or medical status and regardless of their stature prior to expressing a contrary position. In place of open and fair discussion, censorship and suppression of scientific dissent has deleterious and far-reaching implications for medicine, science, and public health.

## Introduction

The emergence of COVID-19 has led to a proliferation of disputes and disagreements over COVID-related knowledge and policy (Liester 2022), including the origin of the SARS-CoV-2 virus (van Helden et al. 2021), restrictive measures taken by most governments such as social-distancing, lockdowns, contact-tracing and mask requirements (Biana and Joaquin 2020), the use of certain treatments of the disease and the exclusion of others (Mucchielli 2020), the safety and efficacy of vaccines against COVID-19, and the implementation of “vaccine passes” in many countries (Palmer 2021). Harambam (2020) has referred to these disputes as the “Corona Truth Wars.”

Since the beginning of the pandemic, while governments and health authorities argued that restrictive lockdown policies were necessary to deal with the pandemic and prevent deaths, many scientists and medical practitioners questioned the ethics and morality of such tactics, including Nobel laureates and leading physicians and scholars (e.g., AIER 2020; Abbasi 2020; Bavli et al. 2020; Brown 2020; Ioannidis 2020a; Lenzer 2020; Levitt 2020). Furthermore, from early 2020, increasing numbers of scientists and doctors argued that the pandemic, as well as morbidity and mortality figures, were being inflated and exaggerated (Ioannidis 2020; Brown 2020); that the extreme policies and restrictions violated fundamental rights (Biana and Joaquin 2020; Stolow et al. 2020); and that governments were using fear campaigns based on speculative assumptions and unreliable predictive models (Brown 2020; Dodsworth 2021). Some scholars, medical practitioners and lawyers have pointed to biases, concealment and distortions of vital information regarding COVID-19 morbidity and mortality rates that misled policymakers and the public (AAPS 2021; Abbasi 2020; AIER, 2020; Fuellmich 2020; King 2020).

It has been argued that much of the discussion around the COVID-19 pandemic has been politicized (Bavli et al. 2020), and that science and scientists are being suppressed due to political and economic interests (Bavli et al. 2020; King 2020; Mucchielli 2020). This criticism has grown, especially following the start of the COVID-19 vaccine campaign. Criticism was made regarding the hastiness with which the mRNA vaccines were granted Emergency Use Authorization by the FDA even for children; the quality of the clinical trials that led to the authorization of the vaccines (including violations of research protocols and evidence of fraud); the lack of transparency regarding the process and data that led to the authorization; the inflation of efficacy estimates; and the minimization or ignoring of adverse events (Doshi 2020, 2021; Fraiman et al. 2022; Thacker 2021).

Critics have argued that the scientific and policy discourse surrounding COVID-19 has not been carried out on a level playing field due to censorship and suppression of views contrary to those supported by medical and government authorities (Cáceres 2022; Cadegiani 2022; Liester, 2022; Mucchielli 2020). Some governments and tech corporations, such as Facebook, Google, Twitter and LinkedIn, have taken measures to censor contrary viewpoints, arguing that views challenging government policies are dangerous misinformation, and therefore censorship is justified to protect public health (Martin 2021).

The present study explores the phenomenon of censorship of dissent from the point of view of well-known scientists and doctors who were censored for their heterodox views on COVID-19, in order to learn about the range of tactics that have been used to censor and silence them, as well as the counter-tactics they have used to resist these attempts.

## Censorship of COVID-19 Heterodoxy

To describe a view or position on COVID-19 as heterodox implies the existence of an orthodox position, which here refers to the dominant position supported by most major governmental and intergovernmental health agencies. Liester (2022) provides a list comparing what he refers to as the dominant versus dissenting views with respect to COVID-19, which includes the origin of SARS CoV-2 (zoonotic vs. laboratory), mask mandates (will prevent spread vs. will not prevent spread), early treatment with drugs such as hydroxychloroquine and ivermectin (ineffective and dangerous vs. effective and safe), the usefulness of lockdown measures and other restrictions (effective and beneficial vs. ineffective and harmful), COVID-19 vaccines (safe and effective vs. unsafe and dangerous), and COVID-19 vaccine mandates and passports (necessary and ethical vs. harmful and unethical). While it may be true that none of these dominant positions have been universally adopted by all governments worldwide to the same degree or down to every last detail, nevertheless a dominant or orthodox position on all of these issues can be identified on a country-by-country basis with strong similarities across national borders.

It is worth noting that orthodox positions can change. For example, by mid-Spring 2020, discussion of the laboratory origins of SARS-CoV-2 was forbidden on certain social media sites, like Twitter and Facebook (Jacobs 2021). More recently the lab-leak theory has since gained more legitimacy, especially following articles in the *Proceedings of the National Academy of Sciences* (Harrison and Sachs 2022), *Frontiers in Virology* (Ambati 2022) and *Vanity Fair* (Eban 2022) as well as a statement by WHO director-general Ghebreyesus, who commented on an interim report by the Scientific Advisory Group for the Origins of Novel Pathogens, saying that all hypotheses need to be considered and criticizing the report for inadequate assessment of the lab-leak hypothesis (WHO 2022). Another example relates to the necessity of mask wearing: US officials such as the director of the National Institute of Allergic and Infectious Diseases (NIAID), Anthony Fauci, are on record recommending against universal mask wearing in March 2020, only to change their position in April to recommend universal mask wearing and mandates (Roche 2021).

Since early 2020, there has been an upsurge of complaints about censorship by individuals and groups presenting heterodox COVID-related viewpoints and information, with even more complaints in 2021 following COVID-19 vaccine rollouts. Many instances involve social media censorship, including the removal of accounts (“deplatforming”) or blocking the visibility of a user’s content without informing them (“shadow banning”) (Martin 2021).

While complaints regarding scientific censorship and suppression preceded the pandemic (Elisha et al. 2021, 2022; Martin 2015), a new feature of the COVID era is the prominent role played by information technology companies such as Facebook and Google (Martin 2021). One prominent example was the down ranking of the Great Barrington Declaration’s website by Google (Myers 2020). The Declaration, spearheaded by three epidemiologists at Harvard, Stanford and Oxford universities, was released in October 2020 (Kulldorff et al. 2020) and signed by many notable scientists and doctors, including the Nobel Prize laureate Michael Levitt. It argued against universal lockdowns in favor of focusing on protecting vulnerable groups. However, to reduce exposure, Google altered its search algorithm (Myers 2020). In February 2021, Facebook deleted a page set up by a group of scientists involved with the declaration (Rankovic 2021). In April 2021, YouTube removed a recording of an official public hearing on the pandemic that featured Florida governor Ron DeSantis and the authors of the Great Barrington Declaration. One of them, Prof. Kulldorff, who is one of the most cited epidemiologists and infectious disease experts in the world, was himself censored by Twitter in March 2021 (Sarkissian 2021). Though his tweet saying that not everyone needs the COVID-19 vaccine was not taken down, he was warned, and users have been prevented from liking or retweeting the post (Tucker 2021).

Similar cases abound. For example, the research-networking site ResearchGate removed physicist Denis Rancourt’s article about masks (Rancourt 2020), and in 2021, it banned him entirely (Jones 2021). In July 2021, LinkedIn suspended the account operated by Dr. Robert Malone, an internationally recognized virologist and immunologist, an action repeated by Twitter in December 2021 (Pandolfo 2021).

These are just some of the many examples of censorship related to COVID-19. Beyond the large scale of the censorship phenomenon, and the wide involvement of tech companies in it, another unique characteristic of COVID-related censorship is its targets. Many of the doctors and researchers being censored by the world’s biggest technology companies are not fringe figures. As in the examples above, these are mainstream scientists, many of them leading experts working in prestigious universities and/or hospitals, some of whom have authored books and published dozens or even hundreds of papers and whose studies have been widely cited. Some of them are editors of scientific/medical journals and some are heads of medical wards or clinics.

This heavy censorship was done with the encouragement of governments (Bose 2021; O’Neill 2021), which cooperated with tech companies such as Facebook, Twitter, and Google. For example, on March 7, 2022, US Surgeon General Vivek Murthy called on tech companies to report “health misinformation” to the federal government and to step up their efforts to remove it (Pavlich 2022). Subsequently, e-mails released from legal proceedings have documented the ways in which government officials directly coordinated with tech companies like Twitter and Facebook to censor doctors, scientists and journalists (Lungariello and Chamberlain 2022; Ramaswamy and Rubenfeld 2022). In December 2021, an e-mail from the fall of 2020 was released via a Freedom of Information Act (FOIA) request. It revealed a behind-the-scenes effort by Francis Collins, then head of the National Institutes of Health (NIH), to his colleague, Anthony Fauci, head of NIAID, to discredit the Great Barrington Declaration and disparage its authors. In the email, Collins told Fauci that “this proposal from the three fringe epidemiologists … seems to be getting a lot of attention,” adding that “there needs to be a quick and devastating published takedown of its premises. I don’t see anything like that online yet—is it underway?” (Wall Street Journal 2021).

Practices of censorship have also been used by the Israeli Ministry of Health (IMOH) and media against doctors and researchers whose views run counter to institutional orthodoxy. One such example is the Israeli Public Emergency Council for the Covid19 Crisis. The organization, which consists of leading doctors and scientists, was targeted by the IMOH and the media numerous times, including attacks on individual members of the organization (Reisfeld 2021).

## Censorship, the Backfire Effect and Public Outrage

COVID-19 censorship is, in part, an exclusion of the views of dissident experts as well as citizens who question the standard position. This type of censorship has been a feature of many other controversial areas in science and medicine, such as AIDS, environmental studies, fluoridation, and vaccination (Delborne 2016; Elisha et al. 2021, 2022; Kuehn 2004; Martin 1991, 1999; Vernon 2017). In fact, censorship has a long history, and its purpose is to suppress free speech, publications and other forms of expression of unwanted ideas and positions that may be perceived as a threat to powerful bodies such as governments and corporations.

Censorship of opposing or alternative opinions and views can be harmful to the public (Elisha et al. 2022), especially during crisis situations such as epidemics, which are characterized by great uncertainties, since it may lead to important views, information and scientific evidence being disregarded. Furthermore, the denial or silencing of contrary views can elicit public mistrust (Gesser-Edelsburg and Shir-Raz 2016; Wynne 2001). Studies have indicated that in situations of risk, especially risk that involves uncertainty, the public prefers full transparency of information, including different views, and that providing it does not raise negative reactions in terms of behaviour, but rather, helps reduce negative feelings and increases the public’s respect for the risk-assessing agency (De Vocht et al. 2014; Lofstedt 2006; Slovic 1994). As Wynne (2001) warns, institutional science’s attempts to exaggerate its intellectual control and use knowledge as justification for policy commitments, while ignoring its limits, only alienates the public and increases mistrust.

Moreover, censorship can be counterproductive, in essence backfiring, because it can lead to greater attention being paid to the censored information, foster sympathy for those being censored and promote public distrust of the actors and agencies engaged in censorship (Jansen and Martin 2003, 2004, 2015). This is especially evident in the internet age. While information technology companies such as Google and Facebook play a prominent role in the attempts of governments and authorities to censor dissenting positions on COVID-19 (Martin 2021), it is a serious challenge to achieve this completely. Their visibility in the mainstream media and in web search results can be curtailed, but there are too many alternative communication options to prevent dissenters from communicating their positions (Cialdini 2016). Therefore, attempts to silence and censor critics can sometimes backfire.

Considering the extent of censorship reported during the COVID-19 era, and in particular the number of accomplished doctors and scientists censored and silenced, as well as the extensive involvement of tech companies, on the one hand, and governments, on the other, it is worthwhile investigating this phenomenon. The present study is designed to explore the subjective perceptions of well-credentialed, highly accomplished mainstream doctors and scientists who have experienced censorship and/or suppression after expressing non-orthodox positions regarding the handling of the COVID-19 pandemic, and how they dealt with it. Through interviews, we examine censorship tactics used by the medical establishment and the media (both mainstream and social media), and the counter-tactics employed by their targets.

## Method

The study is a qualitative one (Aspers 2004), which aims to identify internal perceptions from the point of view of those who have experienced the phenomenon under question.

### Participants

Study participants include 13 established doctors and scientists (12 men and 1 woman), from different countries around the world (viz., Australia, Canada, the Czech Republic, Germany, Israel, UK and US). Of these, 11 have formal medical training from a variety of fields (e.g., epidemiology, radiology, oncology, cardiology, paediatrics, gynecology, emergency room management) and two are research scientists without medical degrees (in the areas of risk management and psychology). All participants hold either an MD or PhD degree, and four hold both. Most of them are well known in their fields, with a proven research background that includes many academic publications. We used a purposeful sampling method, i.e., a non-probabilistic sampling according to which a deliberate selection is made of individuals who could teach us about the phenomenon under study (Creswell 2012). To preserve the respondents’ anonymity, details that might lead to their identification are omitted.

## Research Tool and Procedure

The study is based on in-depth interviews using a semi-structured interview guide. The questions focused on the respondents’ stance towards COVID-19 that was seen as controversial, events they experienced due to their stance, the implications of these events for their professional and personal lives, and their responses to these events.

Recruitment was done in several ways. First, through a Google search we located the contact details of doctors and researchers known for their critical stances toward COVID-19 pandemic measures and policies. Second, we used the “snowball” method to reach other respondents. The initial contact with the respondents was by email, in which we explained the purpose of the study and asked for their consent to be interviewed anonymously. The interviews were conducted via Skype, Zoom or telephone, and lasted about an hour and a half on average. Each respondent was asked to sign an informed consent form. The interviews were recorded and transcribed.

Data analysis and coding were based on identifying the key issues that emerged from the interviews, while classifying and grouping them into meaningful categories. We assured the reliability and validity of the study by applying different methods. The analysis of the data was discussed by all of us as an expert peer group, and different sources of data served as triangulation of the data (e.g., documents and correspondence provided to us by the interviewees). Quotes in the text are provided for illustrative purposes (Creswell 2012).

## Findings

Study participants reported being subject to a wide variety of censorship and suppression tactics used against them by both the medical establishment and the media, due to their critical and unorthodox positions on COVID-19. They also described the counter-tactics they used to resist. We divide the findings into two sections, the first describing censorship and suppression tactics and the second describing the counter-tactics used by our participants.

### Silencing Dissent: Censoring and Suppressing Tactics

Tactics of censorship and suppression described by our respondents include exclusion, derogatory labelling, hostile comments and threatening statements by the media, both mainstream and social; dismissal by the respondents’ employers; official inquiries; revocation of medical licenses; lawsuits; and retraction of scientific papers after publication.

### Exclusion

Respondents reported how, at a very early stage of the epidemic, when they just began to express criticism or their different position, they were surprised to discover that the mainstream media, which until then had seen them as desirable interviewees, stopped interviewing them and accepting opinion pieces from them:

Neither X nor Y [two central newspapers in the respondent's country] wanted to publish my articles. Without a proper explanation. Just stopped receiving articles. It was quite blatant, that they stopped accepting articles expressing a different opinion from that of the ministry of health (MOH). The number of journalists who can really be talked to, who are willing to listen to another opinion, to publish, has been greatly reduced, and most health reporters today are very biased towards the MOH (#10).

### Denigration

Respondents reported that exclusion was only the first step: shortly after that they started being subjected to defamation by the media, and disparaged as “anti-vaxxers,” “Covid deniers,” “dis/misinformation spreaders” and/or “conspiracy theorists”:

After that report came out…, I was front page of the Sunday Times… it said… X [the respondent name], a professor in A [the institution this respondent works in] is co-author of anti vax report… I was now, yeah…, I was told I was anti-vaccine (#9).

I have been vilified.… I’ve been called a quack…, an anti-vaxxer and a COVID denier, a conspiracy theorist (#13).

#### Recruiting “Third Parties” to Assist in Discrediting

One prominent tactic our respondents claim was used by the media to discredit them was the use of seemingly independent “third party sources,” such as other doctors, to undermine them, for example by writing defamatory articles:

I was shocked at what came out the next day in The Wall Street Journal… So here we had three of the most senior doctors with hundreds and hundreds of publications and scientific credibility to our resumes and …a major media outlet allowed a junior doctor to publish who has no academic standing or track record…[and] have him publish a defamatory piece (#6).

Another “third party” source used by the media, according to our respondents, was “fact-checking” organizations, a practice that is ostensibly meant to verify published information to promote the veracity of reporting. However, some respondents alleged that the fact-checking groups were recruited and operated by corporate or other stakeholders to discredit them and try to discredit the information they presented:

…the fact checkers are a source of misinformation, so though it may review something and say, Dr. X said something, but… they make a counterclaim. The counter claims are never cited in the data… they all trace back to the vaccine manufacturers or the vaccine stakeholders (#6).

you get the fact checkers… They tried to discredit S, but also, because I was a co-author, they were picking on me…, and all this sort of stuff and… discredit by association... (#4).

As seen in the second example above, some of the participants said that those “fact-checking” groups were used to discredit and defame not only the researcher or doctor who presented a contrarian opinion or information, but also others who were associated with them.

Some respondents said that the media persecuted them to the point of blackening their name at their workplace, resulting in their dismissal, or that they were forced to resign:

I lost my job..., I was working for the last 20 years in X [the institution’s name]… And so, the media started coming to X… there was a concerted effort to… ruin my reputation, even though, this is unbelievable, they had the lowest death rate basically in the world, and the doctor who brought it to them, gets vilified and slandered. So, I left on my own… My reputation was slandered. I mean the level of treatment that I didn’t expect and abuse I would say (#1).

### Online Censorship

Some respondents reported being censored on social media networks (e.g., Facebook, Twitter, TikTok, YouTube, Google, LinkedIn), and said some of their posts, tweets, videos or even accounts were taken down by the networks.

My YouTube videos were being taken down. Facebook put me in jail, “Facebook Jail.” And I found that I was being de-platformed everywhere (#1).

I’ve always had videos, just my teaching material I’ve been putting up on YouTube…, but I also started to put up materials around this just sort of talking through some of the research… looking at the vaccine efficacy data... YouTube started taking it down. And so now …, I cannot post, I can’t even mention vaccines, because within seconds, as soon as I’m actually trying to upload the video, YouTube will say this video goes against our guidelines... (#3).

I got terminated from TikTok… All of a sudden, I was permanently banned because presumably I had a community violation (#2).

I’m currently on my sixth twitter account…the last one was shut down supposedly for a tweet about X’s lab [the name of the lab], but it was coming. I ruffled too many feathers (#2).

As can be seen in the above examples, respondents noted that the removal of their materials from social networks was accompanied by a notice claiming they had violated the “community rules.” They emphasized that these were academic materials, backed up scientifically:

I became aware that an academic YouTube video that I had put together regarding the paper in the XXX journal … was pulled down by YouTube, and I received a notice that it had violated terms of the YouTube community… without ever having any terms of use from YouTube that would explain what types of terms would be applied to a four PowerPoint slide scientific video…(#6).

One of the respondents reported on censorship even in Google Docs, which means that even private communications are being censored:

Google Docs started restricting and censoring my ability to share documents… This is not Twitter throwing me off like they did. This is an organization telling me that I cannot send a private communication to a colleague or to a friend, or to a family member... (#1).

### Censorship and Suppression by the Medical and Academic Establishment

Some of the respondents reported that they were subjected to defamation by their own institution, with the apparent intention to harm their reputation and careers. For example:

…in [my country], we have approximately 55,000 physicians. My name appeared on the official website of the Ministry of Health, that I’m the only person, one medical doctor who is… distributing disinformation... (#12).

There was a concerted effort to… ruin my reputation even though, this is unbelievable, they [the hospital where I work] had the lowest death rate basically in the world (#1).

Some participants also said that they had received a clear message from the institution where they worked that they were not allowed to identify themselves with the institution when giving an interview or a testimony or expressing their views—in some cases as a condition of renewing their contract.

I gave X (a certain treatment) testimony, and that kind of went viral. And the hospital was not happy because my affiliation had shown up… They offered me a new contract. They said …, we got some new terms for you, because my old contract was not restricted. The new one basically had like seven or eight restrictions of my first amendment rights… basically I couldn’t talk to the press, I couldn’t speak in public…, unless I said, these are my opinions not that of my employer... It was a relatively short conversation. I said that’s never going to happen, I’m never going to sign that thing, and we said goodbye (#9).

In some cases, respondents reported that following a position or criticism they expressed, they were dismissed from their institution, or were notified that their contract would not be renewed.

I was told that my contract [at the medical clinic] wasn’t going to be renewed… There’s a whole variety of checklists for the contract not to be renewed, there must be a due process, and the first red flag is that there was no due process. I asked specifically was there a board vote…, and the answer was no, and I said… why is this action being taken, and their response was “no reason”… [Later] I received a letter from [X] University saying that I was stripped of my professorship, with no due process, with no faculty senate, nothing…. Then, I received a…letter from [Y] University, again no due process, no faculty senate, no explanation (#6).

Similarly, respondents said they were summarily dismissed or disqualified from prestigious positions, such as serving on leading health or scientific committees, or editing medical journals, without due process or transparency:

… the director general of the Ministry of [X] approached me ... and said that the Minister had reached an agreement with the Ministry of Health, that he was putting a representative on the [prescription drug] basket committee ..., and she said all fingers had pointed to me... Then comes a phone call after a week, and she says, “listen, your name was already passed on as a request of the Minister to the Basket Committee, and has been disqualified unequivocally because you oppose [COVID] vaccinations in children”… I was shocked… Until then the responses I received were from the bottom. This is a response from the top (#11).

…there was a whole series of actions taken again with no due process and no explanation so… I received a notice from the [medical association] that I was being stripped from a committee position… I received a letter from a journal…where I was the editor in chief, being stripped of the editorship, again with no due process, no phone calls no, tractable explanation… I received a letter from the National Institutes of Health being stripped from a longstanding committee position, I was on the committee for several decades and was stripped off of that, again no phone call, no due process, no explanation (#6).

In one case, the respondent had learned that his country’s parallel to the Centers for Disease Control (CDC) intervened and asked the university to “examine” his “case”:

…my university president invited me to talk about “corona”. In that meeting, I was informed… that the [The equivalent health authority to the CDC in the interviewees' country] had written a letter to the president, asking him to examine my case because, according to the ministerial letter, I was going public with methodologically questionable things. According to the president, the university has never received similar requests before… (#12).

Some of the interviewees said that the health establishment had not only blackened their reputation and taken serious measures against them but also cooperated with the media and made sure to spread the information about those measures through them:

You know the news release came out, I’m a prominent physician in the United States, so, in fact, I believe the health system drafted a press release that went out, that they were suing me, and so the topic came up [during the press interview], “so are you being sued, and… what’s your reaction?” (#6).

### Official Inquiries

Some doctors reported on official inquiries launched against them, such as investigating or threatening to withdraw their medical license:

…my license was investigated... At that point in time, I felt that the medical board was being weaponized… My license ended up getting investigated… three times now, each time… without any punishment or reprimand or anything... But it merely points to the fact that it’s very easy to get censored or cancelled (#2).

Following a post I wrote about the adverse events ... I received a letter from the Committee of X [the name of the committee]. Allegedly they asked me for the details of these patients (the patients the interviewee reported in his post had adverse events), but if it was genuinely a real wish on their part, then it was not this committee, which in fact deals with Y [the definition of the committee's activities] that would have asked me for the details, but a real official from the Ministry of Health. I answered them through A [my lawyer], a more legal and less medical reply. This is basically a committee without powers. I do not think I am even allowed to pass on the names of patients to them. It can be given to a relevant party, a district doctor… I will be happy to talk to them (#2).

One of the respondents reports that a million-dollar lawsuit was filed against him:

And then my wife calls me and says that the health system is suing us for over a million dollars, so I have just put together teams of attorneys and scrambled them into court... And … the charge is that is that I’m violating terms of my separation agreement, specifically that the health system is being brought into my media presentations, and I’ve never made any [such] statements (#6).

Another respondent reports on a police search conducted at his private clinic in his home:

The [medical board] showed up unexpectedly one day without a warrant to search my house, which was listed in their records as my office, to do a medical office inspection, which doesn’t require a warrant [in my country]. I told them it’s my business office and I don’t see patients there and they have no business coming in there (#7).

### Retraction of Scientific Papers

Some researchers and doctors recounted how their research had been retracted by the journal after publication:

And then, five days before the FDA pediatric meeting on vaccination, [the publishing company] pulls the paper out of the National Library of Medicine and says that they are retracting it. And the explanation, they tell us a few days later, is that they think they didn’t invite the paper to begin with. And I can tell you as an editor, the paper was clearly welcomed, and it went through the standard peer review process. The only way they can legally pull a paper out of the National Library of Medicine is if it’s scientifically invalid, and that wasn’t the claim (#6).

So I submitted it to X [the name of the journal] … and well, this was a desk rejection … Actually, at least for me the arguments were kind of, say, from my perspective, there were no solid arguments… I don't know why this was rejected, and then I submitted it to several other channels... and then I stopped to trying to publish it in the scientific literature. It's published as a pre-print (#8).

Another theme that arose repeatedly during the interviews was that research critical of COVID-19 policies and orthodoxy were treated in ways the interviewees had never encountered before in their careers. This included having papers rejected from journals (often multiple times) without peer review, the journal review and publication process taking many months longer than typical for the journal, and even having papers rejected from pre-print servers such as MedRXiv:

At the beginning of the pandemic, we were getting a lot of stuff published. It wasn’t in any way challenging the orthodox narrative… and then we did this analysis on [X] and then when that happened, oh my God they went ballistic, we got attacked. That work never got published. This is where the censorship—we’d already had some problems because we were publishing other work on the case data, and it was being automatically rejected from any of the medical journals, anything like that. And that was when our stuff started to get rejected from arXiv and medRxiv…the only place we could get any of this stuff published, was we just put it on ResearchGate (#4).

In one case, an interviewee said he felt so threatened by the medical establishment that he refrained from putting his name on papers he co-authored with other researchers, and that those whose names do appear on the papers were trying to hide or stay under the radar until the paper was published:

We’ve got a paper that’s ready to come out in [an important journal], and the group that published it has been hiding for a year... Now, I can’t be on the paper you know (#5).

## Counter-reaction: Fighting Back

The respondents noted that their initial reaction to the attacks and censorship was shock and surprise, since for the first time in their lives they felt excluded from the scientific/medical community, attacked by the media and sometimes by their employers, and/or disparaged as “conspiracy theorists” who endanger the public health. Yet, despite the censorship, the personal attacks and defamation, the dismissals, the damage to reputations and the economic price, all respondents nevertheless stated that none of it deterred them, and they decided to fight back, using various counter-tactics.

### First Reactions: Shock and Surprise

Most respondents describe their initial reaction to the persecution and censorship they experienced as shock. Some said that they felt threatened, and for the first time, excluded from the scientific/medical community:

I was speechless. It does not happen to me. I did not imagine. It was terribly threatening to me all those attacks ... it took me a month to recover from the understanding that this is the country we live in… I was in shock… I was surprised... My heartbeat I think was 200 per minute (#11).

As someone who has been an integral part of the [health] system, and knows the role holders personally - the rift I feel is very heavy (#1).

Respondents said that they felt that the threats, dismissals and attacks against them were in fact an attempt to silence them, just because their opinions were not aligned with those dictated by the authorities:

…everything was done initially to suppress my voice, because I was the only one screaming (#1).

Some respondents said they felt that the censorship and unprecedented attacks they experienced were especially vicious because those who did it knew they were valued and influential:

…they were actually trying to silence me in the media… it appears on the surface, that lawsuit basically was an attempt to censor me… I’m a frequent contributor on Fox News, I just testified in the US Senate…, my advice is valued all over the world, and I think it was a parochial attempt… to censor me…(#6).

### Determined to Fight

Our respondents stated that the censorship and suppression they experienced made them want to fight back and make their voices heard more, on the grounds of freedom of speech and their concern for public health.

It’s an interesting question what I feel I’m paying. Because I feel there are [costs]. The fact is that I almost left. Why did I stay? Because I realized that there was a price that I was not willing to pay - that they would shut me up. (…) (#11).

To me the most important question is why do I (keep) doing this? Because if I do not live according to my values and freedom of speech, then I will not live. That’s why I am doing it (#9).

Some of them even noted that the attacks on their reputation made them even more determined and eager to expose the information that was being censored.

Actually, it makes me more determined. I’m a little bit of a pit bull. So, we’re going to keep getting the word out (#2).

Some of the respondents said they decided to take official or legal actions against the organizations that censored them:

I will file suit for breach of contract, since we had a publication contract and they signed it and they accepted… they’re going to be sued for tortious interference that they’ve actually interfered with the business of publishing valid scientific information... I imagine this is going to be quite injurious and high profile to [the publisher] (#6).

I do have a Freedom of Information request into all the entities that stripped me of various credentials and positions in order to start to uncover what is stimulating all of this... (#2).

The respondents’ counter-reactions were expressed in several ways: a desire to disclose the act of censorship and the information that was censored, which they claim is evidence-based; use of alternative channels in order to spread their positions and views in relation to COVID-19 publicly; establishment of support networks with colleagues; and development of alternative medical and health information systems. That is, they created a kind of a parallel world to the mainstream establishment.

### Exposing the Censorship

Some respondents stressed that they wanted to expose the censorship act itself. For instance:

I got in contact with a few powerful people, and they referred me to the Media Resource Centre in Washington, which is a non-profit to fight censorship. I told them what had happened. And they already wrote up an article about it. That article is now being put up on different sites. I did an interview on One American News Network. I kind of brought that to the world (#1).

### Using Alternative Channels

Respondents noted that when they understood that they were censored by the mainstream media, they decided to use alternative channels, such as social media platforms, to spread their position and contrary information and voice their opinions in public:

Fortunately, I built up a little bit of a Twitter Following… 34,000 or something like that…, so you can get the message out (#4).

Some of the respondents said that to protect themselves, they were forced to open “secret” telegram or anonymous Twitter accounts. Although they express frustration, they are still doing it in order to spread information. For example, one participant noted it is absurd that scientists should keep secret telegram accounts so that the government does not revoke their licenses or damage their reputations:

…my credentials from that aspect [are] really unusual... A working doctor that has that combination… That’s why I have to be careful when I’m on Twitter… because if you’re smart enough to realize there is only a small group of doctors in the world that have got that [combination]… I put a tweet and I put it on my secret telegram channel as well... Ridiculous! We’ve got secret telegram accounts, I mean, we’re scientists running secret telegram accounts, so we don’t get taken out by the government. What is going on? (#5).

### Creating Social Support Networks

Some of the respondents revealed that they created support networks of fellow scientists, physicians, lawyers and politicians with similar views and opinions. These networks were used not only to exchange information, but also to receive support and empathy from “outsiders” like them, to make new friends and create a new community:

…it’s been really nice to make a whole and growing network of friends in life, who know those truths as well. I feel like I'm making a new community with new friends that I can talk to that understand the world, understand the corruption, and really can navigate this stuff. So, at the same time that I woke up with a whole new collection of colleagues and friends, but a lot of us are outside of science... (#9).

And then there were a few colleagues that came on board… And all of a sudden, I had some big heavyweights, academic leaders advocating for my work (#1).

#### Developing Alternative Medical and Health Information Systems

Beyond their activities in disseminating information and data, some of the respondents noted that they are working to establish new alternative platforms and organizations dedicated to developing and providing health information and medical treatments—including new journals and non-profits, instead of the existing ones, which they claim have failed and disappointed. They explain this as a means of coping with the censorship and suppression they experienced due to their opposing positions, which grant them a sense of hope and a feeling that they are building “a new world”:

I have a new thing in life. N and I, we started the X organization…, whose sole mission is to try to figure out and help people to treat COVID. And I think we’ve done a real service to the world (#9).

…there’s a lot of talk about starting a journal… Tess Lawrie started The World Council for Health. There is increasing amount of talk about starting a new health system. Like, people want to go to hospitals where the doctors can be doctors and not the other role of all these regulations and corrupt agencies, so you know, there is maybe a new world that will form…(#4).

## Discussion

The purpose of the present study was to explore the subjective perceptions of accomplished and well-credentialed mainstream doctors and scientists who have experienced censorship and suppression after expressing heterodox COVID-related views, to examine tactics used by the medical establishment and the media and the counter-tactics employed by their targets.

The respondents in our study reported on a wide variety of censorship and suppressive tactics used against them by the media (including the mainstream media and social media companies such as Google, Facebook, Twitter, Instagram, LinkedIn and TikTok) and the medical establishment. Tactics used by the media include derogatory comments and labels, often using ostensibly independent “third-party” sources such as anonymous “fact-checkers” or other doctors, and online censorship involving the removal of their social media and internet contents and accounts, in some cases repeatedly after they opened new accounts. Some of the respondents reported that the media have been persecuting them to the point of blackening their names in their workplaces.

The tactics used by the medical establishment also include defamation and intimidation; retraction of scientific papers after publication; dismissal or adverse changes to employment contracts; aggressive actions aimed at sabotaging other significant roles of the individual, such as participating in important committees or serving as editors of scientific journals. Some respondents reported a targeted thwarting of their careers and harming of the reputations they had built over the years, while consistently stripping them of all the positions they held. Some reported being subject to abusive formal proceedings, such as investigations and attempts to revoke their medical licenses, and in one case even being sued for a large sum of money.

As for the reactions of the respondents to these censorship tactics, contrary to what has been found in previous studies, in which, out of fear of being marked as “anti-science” or “anti-vaxxers,” some of the doctors and scientists said that they refrain from expressing their critical position on controversial issues such as vaccines (e.g., Elisha et al. 2022; Kempner 2008; Martin 2015), the scientists and doctors in our study did not self-censor themselves, despite the heavy price many of them paid professionally and financially. According to the respondents, after the initial shock, they decided to fight back using a range of methods, from framing the actions taken against them as censorship and trying to expose the censored information and the censorship act itself, to mobilizing support and building supportive networks of friends, colleagues, and followers, which, they reported, were constantly growing. Moreover, the respondents announced that they were developing alternative health systems as well as alternative channels that would allow free dissemination of information and professional positions. The sampling method was unlikely to pick up doctors and scientists who kept a low profile or who quickly became silent at the first sign of danger, which may partly explain why all the interviewees resisted attacks. It will also not capture doctors and scientists who disagree with aspects of the official orthodoxy but are too afraid to speak out.

Despite the power held by governments and corporations, the ability to censor is limited, especially in the digital age, since even if the traditional “gatekeepers”—journalists in the popular media and editors of scientific journals—censor opposing opinions and information, opponents will still be able to spread them through alternative outlets. As Jansen and Martin (2003, 2004, 2015) have shown, exposing censorship can sometimes lead to public outrage, and powerful interests who undertake it often try to prevent or reduce this outrage using various methods, mainly by defaming and delegitimizing the targets of censorship.

Indeed, the censorship tactics reported by our respondents are consistent with those identified in Jansen and Martin’s (2015) framework on the dynamics of censorship, including:

1. *Cover-Up*—Our findings show that this tactic was very prominent, which is not surprising, since, as Jansen and Martin noted, if people aren’t aware of censorship, they are not upset about it. The cover-up tactics included various methods. For example, using third-party sources such as other doctors or “fact-checkers” to discredit dissident scientists and doctors. Since these sources are portrayed as independent, they help mask the real sources behind the censorship.
2. *Devaluation*—This tactic was described by our study respondents and included various aspects, such as publishing false and disparaging claims about them, dismissing them from work in academia or medical institutions, and stripping them of various senior positions—all actions that were felt by our respondents to be intended to undermine their credibility and legitimacy. The tactic of devaluation, also known as a “negative campaign” or a “smear campaign,” is often used by corporations, and its aim is to harm the reputation of an individual or a group (Griffin 2012; Lau and Rovner 2009). Smear campaigns help distract public attention from the content of the targets’ message and deflect the discussion from the criticism or allegations raised and instead focus the attention on those raising these allegations.
3. *Reinterpretation*—This tactic involves framing censorship as a means of “protecting the public” from the dissenting doctors and scientists, portraying them as “misinformation spreaders” endangering public health in a time of crisis. This framing echoes attempts by policymakers in other areas to justify censorship by arguing that contradictory information might confuse the public and cause panic (Clarke 2002; Frewer et al. 2003; Sandman 2007; Gesser-Edelsburg and Shir-Raz 2016).
4. *Official Channels*—As our respondents described the censorship actions taken against them were only part of a wider range of silencing and repressive actions, which also included formal proceedings, such as investigating or withdrawing their medical licenses, suing them or ordering a police search of their homes.
5. *Intimidation*—The respondents interpreted all the above tactics as being intended to intimidate and deter them from continuing to publish their views and criticism, and also single them out in a way that implicitly invites harassment by others and serves as an example to other doctors and scientists. Some of our respondents noted they were intimidated to the point they felt it necessary to use an assumed name to continue operating on social media and/or avoid putting their names on papers they co-authored.

Our findings regarding how the study participants responded to censorship tactics are also consistent with the counter-tactics described by Jansen and Martin (2015).

1. *Exposure*—The respondents sought exposure of both the censored information and the censorship itself, for example by raising an alarm about the attacks on them through their social media accounts or other platforms. They noted that even if their accounts were repeatedly removed, they opened new ones or moved to other channels or platforms. In addition, they insisted on continuing to try to publish papers in the scientific literature, regardless of the rejections and retractions, and even if the publication involved working on studies without getting credit for the publication.
2. *Validation*—Our respondents repeatedly stressed their use of evidence-based information and reliable data, as well as their credentials, thus associating themselves with science. They portray themselves as warriors whose mission is to fight against misinformation and censorship by the medical and public health establishment.
3. *Interpretation*—Our respondents framed the media and the establishment’s efforts as censorship and referred to their own efforts as attempts to present valid information for interested readers.
4. *Redirection*—Following the personal and professional attacks they experienced, some of our respondents coordinated a public response, seeking to mobilize their supporters, turning to fellow scientists and doctors, and creating alliances and cooperation networks.
5. *Resistance*—Despite the initial shock, all the respondents said they decided not to succumb, but rather to resist and fight back.

Our findings echo arguments made in previous studies on the suppression of dissent in controversial areas, such as vaccination (Elisha et al. 2021, 2022; Cernic 2018; DeLong 2012; Gatto et al. 2013; Martin 2015; Vernon 2017), AIDS, environmental studies, and fluoridation (e.g., Delborne 2016; Kuehn 2004; Martin 1981, 1991, 1999). Similar to those studies, our research findings indicate significant involvement of the media and the medical establishment in censorship and suppression of dissenters.

Yet, there are three main differences. First, when it comes to COVID-related knowledge, the censorship tactics used against dissenters are extreme and unprecedented in their intensiveness and extensiveness, with scientific journals, and academic and medical institutions taking an active and involved part in censoring critical voices. In fact, as one of our respondents indicates, even pre-print servers and academic social networking sites censor scientific papers that do not align with the mainstream narrative, and this seems to be a growing trend. One recent example is a study report by Verkerk et al. (2022), which analyzed a survey of over 300,000 people in 175 countries who had elected to not receive COVID-19 vaccines, which was removed from ResearchGate.net after 9 days citing a breach of their terms and conditions (World Council for Health 2022). Furthermore, what our respondents describe goes way beyond censorship, and includes a wide range of suppression methods intended to destroy their reputations and careers, solely because they dared to take a different position from that dictated by the medical establishment.

Second, while previous studies have also indicated isolated cases where researchers and doctors with flawless résumés and even senior academic or medical status were censored if they dared express dissenting opinions, the current study shows that in the case of COVID, censoring doctors and researchers of this stature has become a regular phenomenon. The participants in our study, as well as those mentioned in the introduction and many others not included in our sample, are not fringe scientists. Most of them are leading figures: researchers and doctors who prior to the COVID-19 era had a respectable status, with many publications in the scientific literature, some of them with books and hundreds of publications, some headed academic or medical departments, some of them were editors of medical journals, and some had won significant awards. Nevertheless, as our findings show, they were not protected from censorship, nor from the suppression and defamation campaign launched against them. This fact indicates that the message is that no one is exempt from censorship and no academic or medical status, senior as it may be, is a guaranteed shield against it.

The third prominent difference found in our study is the significant role played by media organizations during the COVID pandemic, and especially tech information companies, in censoring contrary positions. On a practical level, those who hold the power have greater ability and opportunities to control knowledge and information dissemination, and through this, to set and control the agenda. While our findings do not show the direction of the relationship between these interest holders, they may indicate collaborations between the medical establishment and these companies. Recently released documents from court cases indicate that at least some of this censorship is orchestrated by government officials (Lungariello and Chamberlain^2022^; Ramaswamy and Rubenfeld 2022). Our findings also indirectly point to other stakeholders involved in the censorship phenomenon evident in the current crisis, especially pharmaceutical companies. While our study examined the subjective perceptions of those targeted by censorship rather than the involvement of stakeholders and other interested parties, our respondents’ reports echo findings from other studies, conducted both prior to the COVID-19 era (Ravelli 2015), and more recently (Mucchielli 2020), which indicate the extensive involvement of pharmaceutical and information tech corporations in silencing information and studies that may be unfavourable to them. Given the central role of these corporations alongside policymakers in health authorities and governments globally, a major concern is that substantial interests, including financial and political ones, as well as interests related to reputation and career, may lay behind the suppression efforts. The interest of the pharmaceutical corporations in controlling the discourse regarding COVID-19 is self-evident. For example, as some of our participants indicated, one of the main unresolved COVID-19 controversies is related to early treatment with repurposed drugs, and it has been claimed that highly unusual measures were taken to prevent physicians from using them (Physicians’ Declaration 2021). As Cáceres (2022) notes, this alleged unwarranted termination of that initial debate may have had enormous economic (e.g. green light for vaccines and new drugs under emergency use authorization), financial (e.g. huge gains for the largest corporations) and political consequences (e.g. global restrictions of individual freedoms).

The tech information companies also have strong interests in controlling the discourse regarding the COVID-19 pandemic. For example, in June 2021, it was revealed that Google, which was accused of silencing the theory the SARS-CoV-2 virus leaked from the Wuhan Institute of Virology, has funded virus research carried out by a Wuhan-linked scientist, Peter Daszak, through its charity arm, Google.org**,** for over a decade. Google has also invested one million dollars in a company that uses epidemiologists and big-data analytics to forecast and track disease outbreaks. The *British Medical Journal* has revealed that Facebook and YouTube’s “fact-checking” process relies on partnerships with third-party fact-checkers, convened under the umbrella of the International Fact-Checking Network (Clarke 2021). This organization is run by the Poynter Institute for Media Studies, a non-profit journalism school whose main financial supporters include Google and Facebook.

As for policymakers’ personal interests, a US government watchdog group has been demanding key data on Dr. Anthony Fauci's financial and professional history, claiming that “During the pandemic, Dr. Fauci has handsomely profited from his federal employment, royalties, travel perks, and investment gains,” yet it is not public what his salary was during these two years, nor what stocks and bonds he bought and sold in 2020 or 2021, as he influenced COVID policies, or what he received—or didn’t receive—in royalties. As noted earlier, a FOIA request in the US revealed that Fauci was told by Francis Collins, then head of the NIH, to discredit the Great Barrington Declaration and disparage its authors (Wilson 2021). Roussel and Raoult (2020) found similar conflicts of interest among French doctors who took a public stand against the use of hydroxychloroquine.

Censorship undermines public trust in authorities, especially if the information hidden and later on revealed might have cost human lives, such as during pandemics, which involve diseases, treatments and vaccines (Gesser-Edelsburg and Shir-Raz 2018). In addition, censorship and manipulation of information are inconsistent with the essence of science, since scientific inquiry requires discourse and vigorous debate. Indeed, researchers have warned that instead of being debated, COVID controversies are being used to fuel polarization, often leading to the demonization and censorship of alternative perspectives and the imposition of mainstream views as if they were absolute truth (Cáceres 2022; Marcon and Caulfield 2021).

Cáceres (2022) has argued that the fact that the debate was silenced and alternative positions were censored is in fact a diversion from “normal science” (Kuhn 1962), which assumes that different explanations and answers to facts of scientific interest normally emerge, and have the opportunity to be resolved in conventional scientific debate. Such diversion from “normal” scientific praxis, Cáceres maintains, suggests that “non-scientific” influences are at work. This diversion is especially concerning when the voices silenced are those of a mounting number of leading and renowned scientists and doctors. The drive to censor and dismiss dissenting opinions by labeling them as “misinformation” shares close similarities with scientific “boundary work,” wherein scientific power and authority is maintained by demarcating certain fields of scientific inquiry as out of bounds and discrediting them as essentially unscientific (Gieryn 1999; also see Harambam 2014). Creating a false consensus by censoring information and preventing scientific debates might lead scientists, and thus also policymakers, to sink into the ruling paradigm, causing them to ignore other, more effective options to cope with the crisis or perhaps even prevent it. Such a “consensus” leads to a narrow worldview, which impairs the public’s ability to make informed decisions and erodes public trust in medical science and in public health (Cernic 2018; Delborne 2016; Martin 2014, 2015; Vernon 2017).

The main limitation of the study is that the findings are based on the subjective perspectives of interviewees. It is possible that if we included more heterogeneous groups, we would come to somewhat different interpretations. Therefore, we recommend conducting further studies among larger groups of professionals who suffered censorship, to expand our knowledge and perhaps suggest effective ways to mediate the struggle over freedom of information in general and especially in times of crisis.

One main contribution of this study is in giving voice to scientists and doctors who raise questions, doubts or criticism in controversial areas in public health and science, especially during times of crisis. At the same time, we seek to raise awareness of the increasing use of censorship practices and aggressive tactics of suppression, targeting even leading figures who dare to criticize or doubt the dictated “consensus.” Censorship and silencing practices can have far-reaching consequences, manifested in the violation of freedom of speech and of ethical principles, harming science, and potentially risking public health and safety (Elisha et al. 2022). Researchers have already warned that the COVID-19 crisis confirms previous concerns about the deleterious implications of censorship (Cáceres 2022; Mucchielli 2020). We concur with Cáceres’ assertion that censorship and dogma are foreign to true science and must be abandoned and replaced by open and fair discussion.
